# Cyanosis in the Fatal Broncho-Pneumonia of Influenza
*Part of an Essay for which a Martyn Memorial Pathological Scholarship was awarded, 1931.


**Published:** 1931

**Authors:** Frank E. Fletcher


### CYANOSIS IN THE FATAL BRONCHO-PNEUMONIA OF INFLUENZA.*

BY

Frank E. Fletcher.

---

After an historical survey of the causation and pathology of influenzal broncho-pneumonia, the essay is devoted to a consideration of the causes of cyanosis in that disease, and proceeds:

In the bad cases of influenzal broncho-pneumonia one of the symptoms is a peculiar blue or violet cyanosis. J. S. Haldane[1] observes: "The cyanosis was attributed to the broncho-pneumonia, but against this explanation was the fact that the administration of oxygen failed to abolish or decrease the cyanosis, whereas the cyanosis usually accompanying broncho-pneumonia can be relieved or abolished easily by adding some oxygen to the inspired air." He suggested, on the evidence that he gave, that the hæmoglobin was in the form of methæmoglobin, and that this was converted post-mortem to nitric-oxide hæmoglobin. Sadie[2] showed this to be improbable, inasmuch as he demonstrated that, although there was a marked oxygen desaturation of arterial blood, it was not due either to methæmoglobin or sulphæmoglobin.

The gases in the arterial blood in several cases have been estimated, and it was seen that there was

* Part of an Essay for which a Martyn Memorial Pathological Scholarship was awarded, 1931.

a definite oxygen desaturation of the arterial blood. It would seem probable that the factors producing this great oxygen want were of such a nature as to allow large quantities of venous blood to return to the left side of the heart and so pollute the general circulation. It was seen that the carbon-dioxide content of the arterial blood in these cases was not diminished, not even to any great extent with the administration of oxygen. It has been suggested that this indicates that there was a condition present which prevented carbon-dioxide from being removed from a considerable number of alveoli in spite of increased ventilation. In other words, there were parts of the lung through which the circulation continued more or less freely, but the alveoli of these areas were potentially cut off from being ventilated under existing conditions. That the isolation of these areas may sometimes be incomplete was indicated by the fact that, if the partial pressure of oxygen in the inspired air was sufficiently high, the air in such alveoli was eventually enriched and supplied the blood with oxygen. It was noticed, too, that where the arterial oxygen saturation and carbon-dioxide content were estimated, there was parallelism between the oxygen desaturation and carbon-dioxide content of the arterial blood ; the greater the former the greater the latter.

As the disease progressed there was a decline in the carbon-dioxide combining powers of the blood which was coincident with definite signs of marked consolidation of large areas of lung tissue. This would produce more or less complete cessation of circulation through these areas, and the venous blood returning to the general circulation would be less abundant.

The conspicuous change in the carbon-dioxide dissociation curve is that there was a very high curve in the early stages, and as the disease advanced there was a progressive decline in the carbon-dioxide combining power of the blood, which never reached the normal curve. This has been explained by the occurrence, in the first instance, of a pronounced gaseous acidosis, which has been more or less compensated for by an increase in the alkaline reserve. It has also been determined that, whatever the oxygen saturation of the arterial blood in this disease, the dissociation curve is almost normal. It therefore seems clear that the oxygen want is directly the result of interference with the respiratory function in the lungs. It has been suggested that two mechanisms might lead to such interference. Firstly, areas of the lobules of the lung have been rendered non-functional by the closure of bronchioles without pronounced damage to the alveoli, and here the circulation will continue to a certain extent. The partial pressure of oxygen here, in the cut-off alveoli, will be rapidly reduced, while the carbon dioxide content will not be increased appreciably beyond an equilibrium with that of the venous blood. Hence numerous small areas of lung will be non-functional, although a certain amount of circulation is still possible. For instance, if a given amount of blood is circulating in a lung in which 50 per cent. is not working yet allows circulation, the blood only takes up 50 per cent. of its normal oxygen ; therefore, venous blood will be returned to pollute the mixed blood.

The other mechanism leading to oxygen want is that of rapid and shallow breathing, which increases the relative dead space and, in addition, must give uneven ventilation of the pulmonary alveoli.

The cyanosis of carbon - dioxide retention is plum-coloured. In rapid and shallow breathing there would be expected a deficiency of carbon-dioxide in the arterial blood proportional to the oxygen want, and this would lead to a pale greyish cyanosis ; while the leaden grey to heliotrope type of cyanosis, met with in influenzal broncho-pneumonia, suggests a combination of the two.

In the following three cases of influenzal broncho-pneumonia the blood, withdrawn post-mortem, was examined spectroscopically.

**Case 1.** — Housewife, aged 51.

*Habitat.*—Unhealthy basement flat in healthy neighbourhood.

*History.*—Symptoms of influenza for about seven days. Then signs of explosive crepitation over base of left lung, and in patches over right lung. Next day dirty azure-blue cyanosis. Oxygen administered with very little improvement. Patient died on tenth day from onset of fever.

*Post-mortem.* — A well - nourished individual having at least two inches of subcutaneous fat. The lips still showed signs of cyanosis. The heart was enlarged and very fatty, weighing 22 oz., while there was fatty infiltration deeply into the muscle walls. A small quantity of blood was removed for examination. Over the whole of the posterior portion of the right lung and over the lower lobe were recent adhesions. Recent adhesions were also present posteriorly over the left lung. No definite areas of depression were seen, but the right lung felt soft and fleshy, with small areas which were more resistant, but the lung was still crepitant. On section it was seen to be the seat of hæmorrhagic œdema, and small areas of darker colour could be seen. Muco-purulent secretion was squeezed out of the bronchioles. Smears were made from this. The left lung showed the same changes as the right, only not as markedly. The trachea and bronchi contained muco-purulent secretion and showed signs of a recent bronchitis. The tracheo-bronchial glands were normal. The smears showed all Gram-positive organisms ; a few staphylococci, but a far greater amount of streptococci, in chains varying from five to eight cocci in length. There were also present a few cells undergoing degenerative changes ; these were thought to be desquamated epithelial cells, and in some less changed cells cocci resembling isolated streptococci were seen. The blood was examined with a spectroscope, and showed the oxyhæmoglobin bands. It was also easily reduced to hæmoglobin. There were no signs of either methæmoglobin or nitric-oxide-hæmoglobin.

**Case 2.**—Male tramp, aged 45.

*Habitat.*—Casual wards.

*History.* — Had had a "cold" for about ten days. Complained of feeling giddy and faint.

Sat down by side of road and died. Police constable who saw him immediately after death said his lips were dark blue.

*Post-mortem.*—An extremely emaciated subject. There were no fat reserves anywhere; the omentum was thin and transparent. There were signs of mesaortitis in the arch of the aorta and the orifices of both coronary arteries were partially occluded. Patchy fibrosis of heart muscle was present. There were recent pleural adhesions over the left lung, but not on the right side. The left lung presented the same appearances as the right lung of the preceding case. The right lung, although no definite areas of consolidation could be felt, appeared to be more airless and hæmorrhagic than usual. The trachea and bronchi showed the same changes as Case 1. The tracheo-bronchial glands were normal. Smears and blood were taken as before. Almost the only organisms present in the smears were medium length streptococci, the majority varying from five to ten cocci in length. A spectroscopic examination of the blood showed the bands of oxyhæmoglobin only; no methæmoglobin was detected.

**Case 3.**—Publican, aged 47.

*Habitat.*—Slum area.

*History.*—"Feverish cold" for about five days. Found dead in beer cellar. The doctor who saw him immediately after death said his lips and neck were "slatish blue in colour."

*Post-mortem.*—A well nourished man, still showing signs of cyanosis. The right heart was very congested, the mitral valves were considerably thickened and showed that there must have been some regurgitation of blood during life. A sample of blood was taken. Excepting that it was the right lung which was chiefly affected, the lungs were almost identical with the last case. The trachea and bronchi contained a fair

S

quantity of muco-purulent pus, and atrophy of the mucous membrane, the cartilaginous rings being very well marked. The tracheo-bronchial glands were slightly enlarged and soft. The smear from the exuding bronchioles showed numerous streptococci in chains of three to ten cocci in length; occasionally they seemed to take a diplococcal form while in the chains. Several were seen either in or on desquamated epithelial cells, but their staining was not affected. There were also a small number of diplococci present, of smaller size than the streptococci. The blood again did not show the presence of methæmoglobin, but in this case the spectrum in a more concentrated solution showed the lines of hæmoglobin. In reducing the oxyhæmoglobin with ammonium sulphide care had to be taken, as a temperature much below the stated one—that of 50° C.—was sufficient to convert methæmoglobin into oxyhæmoglobin, and eventually into hæmoglobin.

These observations support the view that the cyanosis is due to (1) much of the blood circulating round non-functioning alveoli; (2) uneven ventilation of the functioning alveoli, brought about by increase of dead space. Fatal results are liable to occur suddenly with an already diseased heart when there is congestion in the pulmonary circulation, accompanied, as it must be, by lack of oxygen supply to its tissues.

My thanks are due to the Acting Police Surgeon of Bath, Mr. E. Scott White, for allowing me to perform the autopsies and to use his materials and laboratory.

REFERENCES.

[1] *Brit. Med. Jour.*, 1929, i. 1,070.

[2] *Jour. Exp. Med.*, 1919, xxx. 215.

